# Lineage-Dependent Regulation of Glutathione Homeostasis by EAAC1 and GTRAP3-18 During Differentiation of Mesenchymal Stem Cells into Neuron-like Cells

**DOI:** 10.3390/ijms27125323

**Published:** 2026-06-12

**Authors:** Nobuko Matsumura, Wattanaporn Bhadhprasit, Koji Aoyama

**Affiliations:** Department of Pharmacology, Teikyo University School of Medicine, 2-11-1 Kaga, Itabashi, Tokyo 173-8605, Japan; matsumura.nobuko.bt@teikyo-u.ac.jp (N.M.); sumida.wattanapon.ck@teikyo-u.ac.jp (W.B.)

**Keywords:** EAAC1, GTRAP3-18, glutathione metabolism, transporter trafficking, mesenchymal stem cells, neuronal differentiation

## Abstract

Adult bone marrow-derived mesenchymal stem cells (BMSCs) are multipotent progenitors capable of differentiating into diverse cell lineages, including osteogenic, chondrogenic, adipogenic, and neuronal lineages. In BMSCs, intracellular glutathione (GSH) is a critical determinant of stemness maintenance and differentiation outcomes. However, how intracellular GSH homeostasis is regulated during BMSC-to-neuron differentiation remains unclear. In neurons, GSH synthesis critically depends on cysteine uptake mediated by the excitatory amino acid carrier 1 (EAAC1). Here, we investigated the expression, subcellular localization, and functional contribution of EAAC1 and its regulatory protein, glutamate transporter-associated protein 3-18 (GTRAP3-18) in mouse BMSCs and neuron-like BMSCs generated by Notch intracellular domain-based induction (NICD-3F BMSCs). BMSCs exhibited higher intracellular GSH levels than NICD-3F BMSCs, despite comparable levels of EAAC1 protein. In contrast, EAAC1-dependent cysteine uptake and plasma membrane localization of EAAC1 were markedly reduced in BMSCs, indicating differentiation-dependent regulation of EAAC1 trafficking. Treatment with the xCT inhibitor erastin reduced intracellular GSH levels in both BMSCs and NICD-3F BMSCs. GTRAP3-18 expression was high in BMSCs and significantly reduced in NICD-3F BMSCs. Notably, GTRAP3-18 knockout decreased intracellular GSH levels in BMSCs without altering total EAAC1 protein or intracellular cysteine levels, whereas in NICD-3F BMSCs, both GSH and EAAC1 protein levels were increased. These findings demonstrate lineage-dependent divergence in GSH regulatory mechanisms and reveal previously unrecognized functions of GTRAP3-18 in redox control during stem–to–neuron differentiation.

## 1. Introduction

Maintaining intracellular redox balance is essential for cell survival, differentiation, and resistance to oxidative stress. Among cellular antioxidant systems, glutathione (GSH) represents the most abundant and versatile redox buffer, regulating detoxification of reactive oxygen species, protein thiol redox state, and metabolic homeostasis [1,2]. Cellular GSH levels are largely determined by the availability of cysteine, the rate-limiting substrate for GSH synthesis. Cysteine can be supplied through multiple pathways, including proteolysis, methionine metabolism via the transsulfuration pathway, and uptake from the extracellular environment, with the relative contribution of each pathway varying in a cell-type-specific manner [3,4].

Bone marrow-derived mesenchymal stem cells (BMSCs) require robust intracellular redox regulation to maintain their survival, proliferative capacity, and functional plasticity [5,6,7]. Although the cystine/glutamate antiporter xCT (system Xc^−^) has been implicated as one contributor to cystine supply and GSH synthesis in BMSCs [8,9], studies addressing redox-regulating mechanisms in these cells remain limited. Consequently, it is still unclear how BMSCs acquire cysteine—the rate-limiting substrate for GSH biosynthesis—under physiological conditions or in response to oxidative stress, and whether additional cysteine transport systems contribute to GSH homeostasis in stem cells.

In neuronal cells, by contrast, de novo GSH synthesis is known to depend critically on extracellular cysteine uptake mediated by the high-affinity transporter excitatory amino acid carrier 1 (EAAC1; also known as EAAT3 or SLC1A1) [10,11,12,13,14]. EAAC1 plays a central role in maintaining neuronal GSH levels and is tightly regulated by subcellular trafficking between the endoplasmic reticulum (ER) and the plasma membrane [15,16,17]. A key negative regulator of this trafficking is the ER-associated protein glutamate transporter-associated protein 3-18 (GTRAP3-18, also known as ARL6IP5), which binds EAAC1 and restricts its surface delivery, thereby limiting cysteine uptake and GSH synthesis [18,19,20]. Importantly, neuronal development and tissue remodeling in both embryonic and adult organs rely on tightly regulated GSH metabolism [1,21], underscoring the physiological significance of EAAC1-dependent redox control in neural lineages [22]. Consistent with this notion, loss of GTRAP3-18 has been shown to increase neuronal GSH levels, confer neuroprotection, and promote adult hippocampal neurogenesis, suggesting that GTRAP3-18 may modulate redox balance and neurogenesis in neural lineages [23,24].

BMSCs possess the capacity to differentiate into neuron-like cells, providing an experimental framework to examine how redox regulatory mechanisms are remodeled during stem-to-neuron lineage transition. As a clinically relevant adult stem cell population, BMSCs provide a suitable model to investigate mechanisms regulating intracellular redox balance and GSH homeostasis in the context of stem cell function. Despite the well-established role of EAAC1 in neurons, the contribution of EAAC1 to cysteine acquisition and intracellular GSH homeostasis in undifferentiated BMSCs has not been evaluated. Moreover, it remains unknown whether the EAAC1–GTRAP3-18 regulatory axis, which is critical for neuronal redox balance, operates in BMSCs or is selectively engaged during neuronal differentiation.

In the present study, we investigated the expression, subcellular localization, and functional contribution of EAAC1 and GTRAP3-18 in mouse BMSCs and neuron-like cells generated by the Notch intracellular domain (NICD-3F BMSCs). By integrating biochemical analyses of intracellular GSH levels, assessment of cysteine uptake activity, evaluating the effects of EAAC1 inhibition, subcellular localization studies of EAAC1, and genetic deletion of GTRAP3-18, we demonstrate that redox regulation diverges in a lineage-dependent manner. Our findings reveal that EAAC1 trafficking and activity become increasingly important following neuron-like differentiation, whereas GTRAP3-18 contributes to GSH homeostasis in BMSCs through mechanisms that are largely independent of EAAC1-mediated cysteine uptake. These results uncover a previously unexplored metabolic axis linking cysteine availability, transporter regulation, and redox balance during stem-to-neuron differentiation, and provide new insight into how BMSCs manage oxidative stress and acquire neural characteristics.

## 2. Results

### 2.1. Characterization of Isolated BMSCs

**Figure 1:**
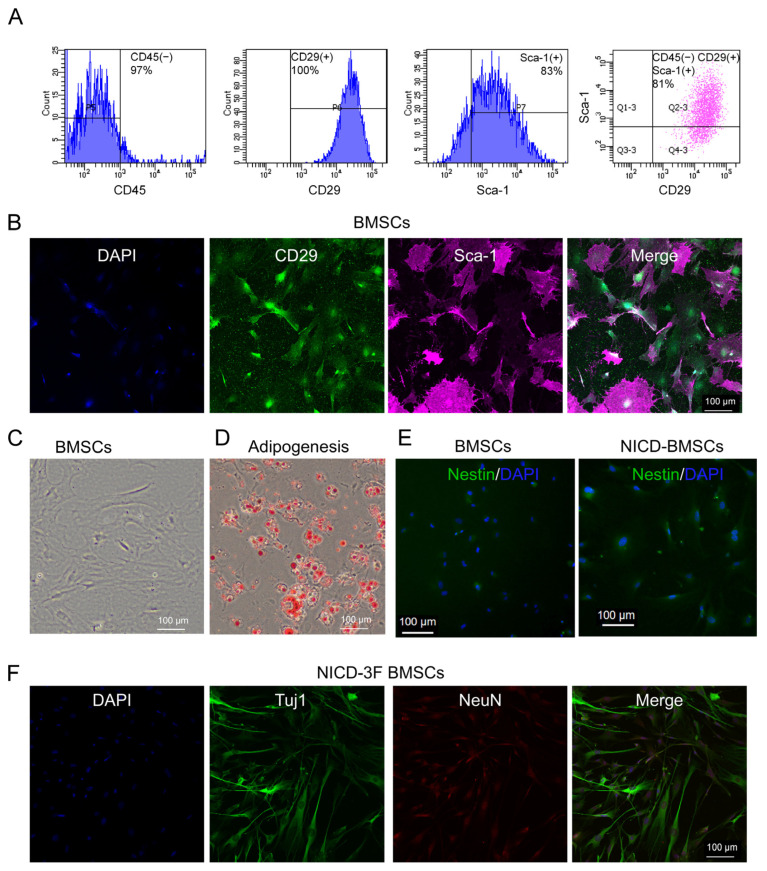
Phenotypic characterization and multipotent differentiation capacity of mouse bone marrow-derived mesenchymal stem cells (BMSCs). (**A**) Flow cytometric analysis of cultured mouse BMSCs showing staining for CD45, integrin β1 (CD29), and stem cell antigen-1 (Sca-1). The majority of the population was CD45^−^ and CD29^+^/Sca-1^+^, accounting for approximately 81% of total cells. Percentages of each gated population are indicated in the plots. (**B**) Immunocytochemical staining demonstrating expression of CD29 (green) and Sca-1 (magenta) in BMSCs. (**C**) Bright-field image of BMSCs exhibiting an adherent fibroblast-like, spindle-shaped morphology. (**D**) Adipogenic differentiation visualized by Oil Red O staining. (**E**) Nestin expression in BMSCs following electroporation-mediated introduction of the Notch intracellular domain (NICD) gene (NICD-BMSCs). Nestin staining is shown in green, with DAPI in blue. (**F**) Neurogenic differentiation of BMSCs. NICD-BMSCs were treated with forskolin (FSK), basic fibroblast growth factor (bFGF), and ciliary neurotrophic factor (CNTF), resulting in neuron-like cells (NICD-3F-BMSCs) expressing βIII-tubulin (Tuj1, green) and NeuN (red). Scale bars represent 100 µm.

Flow cytometric analysis of BMSC markers showed that mouse BMSCs expressed the mesenchymal stem cell (MSC) markers integrin β1 (CD29) and stem cell antigen-1 (Sca-1), and lacked the hematopoietic marker CD45. CD29^+^/Sca-1^+^/CD45^−^ cells accounted for approximately 81% of the total culture (Figure 1A). Immunostaining also showed that the majority of cells were positive for both CD29 and Sca-1 (Figure 1B). A subset of cells with weaker or absent Sca-1 staining was also observed, consistent with the expected purity of mouse BMSC preparations. The cells displayed adherent growth with a characteristic fibroblast-like, spindle-shaped morphology (Figure 1C). In addition, adipogenic differentiation was confirmed by the presence of Oil Red O-positive lipid granules (Figure 1D). These findings demonstrate that the isolated cells retain hallmark MSC phenotypes and multipotent differentiation capacity suitable for use as an adult stem cell model in this study.

### 2.2. Characterization of Neuron-like Cells Derived from BMSCs

Neuron-like cells were generated via NICD-based induction. NICD signaling is reported to promote neural stem/progenitor-like properties, including upregulation of nestin [25]. Electroporation-mediated introduction of NICD increased nestin immunoreactivity in BMSCs compared with untreated cells (Figure 1E). Subsequent treatment with forskolin (FSK), basic fibroblast growth factor (bFGF), and ciliary neurotrophic factor (CNTF) for 3–5 days induced neuronal differentiation (NICD-3F-BMSCs). These neuron-like cells expressed βIII-tubulin (Tuj1) and NeuN (Figure 1F), confirming successful induction of neurogenic features. Collectively, these data support the use of BMSCs and NICD-3F BMSCs as an in vitro system to model adult stem cell-derived neurogenesis in subsequent analyses. However, microtubule-associated protein 2 (MAP2) expression was weak in NICD-3F BMSCs, indicating that these cells represent a partially differentiated neuronal state rather than fully mature neurons (Supplementary Figure S1A).

### 2.3. Intracellular GSH and EAAC1 Function in BMSCs and NICD-3F BMSCs

**Figure 2:**
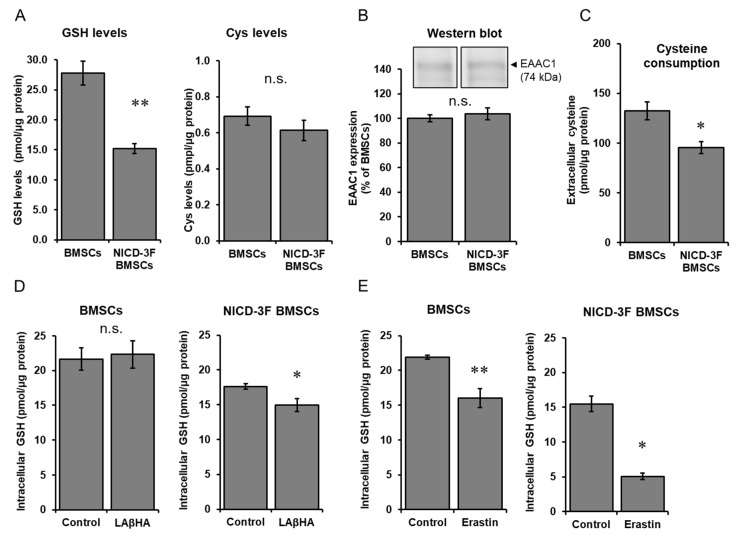
Intracellular glutathione (GSH) metabolism and excitatory amino acid carrier 1 (EAAC1) function in BMSCs and neuron-like differentiated BMSCs (NICD-3F BMSCs). (**A**) Quantification of intracellular cysteine and GSH by High-performance liquid chromatography (HPLC). NICD-3F BMSCs (*n* = 15) showed significantly lower GSH levels than BMSCs (*n* = 17), while cysteine levels were comparable. (**B**) Western blot analysis of EAAC1 expression in BMSCs and NICD-3F BMSCs. Total EAAC1 protein levels did not differ significantly between BMSCs and NICD-3F BMSCs (*n* = 3). (**C**) Cysteine uptake activity assessed by measuring the remaining extracellular cysteine 90 min after supplementation. A greater reduction in extracellular cysteine was observed in NICD-3F BMSCs (*n* = 3). (**D**) Effect of the EAAC1 inhibitor L-aspartic acid β-hydroxamate (LAβHA) on intracellular GSH levels. LAβHA reduced GSH in NICD-3F BMSCs (*n* = 6) but had minimal effect in BMSCs (*n* = 3). (**E**) Effect of the xCT inhibitor erastin on intracellular GSH levels. Erastin reduced intracellular GSH levels in both BMSCs (*n* = 7) and NICD-3F BMSCs (*n* = 3). For panel (**A**), data were obtained from four independent mice, with 3–6 technical replicates per mouse (*n* = 15–17). For panels (**B**,**C**), as well as BMSCs in panel (**D**) and NICD-3F BMSCs in panel (**E**), *n* = 3 represents technical replicates derived from the same initial cell population. For NICD-3F BMSCs in panel (**D**), and BMSCs in panel (**E**), data were obtained from 2 to 3 independent mice, each with 1–4 technical replicates (*n* = 6–7). Data are presented as means ± s.e.m. * *p* < 0.05; ** *p* < 0.01 compared with BMSCs or vehicle control, as indicated. n.s., not significant.

To investigate how GSH metabolism changes during neuronal differentiation, intracellular cysteine and GSH concentrations were measured by High-performance liquid chromatography (HPLC). BMSCs contained significantly higher GSH levels than NICD-3F BMSCs, whereas intracellular cysteine levels did not differ between the two groups (Figure 2A). The GSH concentration in BMSCs was approximately 1.7-fold higher than in neuron-like differentiated BMSCs. This result is similar to the 1.5-fold higher GSH levels reported in astrocytes compared with neurons [26]. EAAC1-mediated cysteine uptake is a major determinant of neuronal GSH synthesis. We examined total EAAC1 protein expression in both BMSCs and NICD-3F BMSCs to examine the difference in the role of EAAC1 on GSH synthesis between the two cell types. Western blot analysis revealed no significant difference in 74 kDa EAAC1 abundance between BMSCs and NICD-3F BMSCs (Figure 2B). Total EAAC1 protein levels (74 kDa) did not differ significantly between BMSCs and NICD-3F BMSCs (Figure 2B).

To assess functional EAAC1 activity, cysteine uptake was estimated from the decrease in extracellular cysteine 90 min after supplementation. NICD-3F BMSCs exhibited a greater reduction in extracellular cysteine than BMSCs, indicating higher cysteine consumption despite comparable EAAC1 protein levels (Figure 2C). We next tested whether EAAC1 contributes to intracellular GSH under these conditions. Treatment with the EAAC1 inhibitor LAβHA significantly reduced GSH levels in NICD-3F BMSCs (~16.1%) but had minimal effect on GSH levels in BMSCs (Figure 2D). LAβHA had no significant effect on intracellular cysteine in either group (Table S1), consistent with rapid incorporation of imported cysteine into GSH.

To further investigate the mechanisms underlying GSH regulation, we examined the effect of the xCT inhibitor erastin (Figure 2E). Treatment with erastin significantly reduced intracellular GSH levels in BMSCs. A similar reduction in GSH levels was also observed in NICD-3F BMSCs, with a more pronounced decrease compared to BMSCs. Erastin had no significant effect on intracellular cysteine levels in either group (Table S2). No significant cytotoxic effects were observed for either LaβHA or erastin under these conditions, as indicated by unchanged total protein yield per well.

Together, these results suggest that EAAC1 activity contributes to GSH maintenance in NICD-3F BMSCs and that multiple transport mechanisms may be involved, whereas GSH regulation in BMSCs appears to be less dependent on EAAC1 function.

### 2.4. Plasma Membrane Translocation of EAAC1 in BMSCs and NICD-3F BMSCs

**Figure 3:**
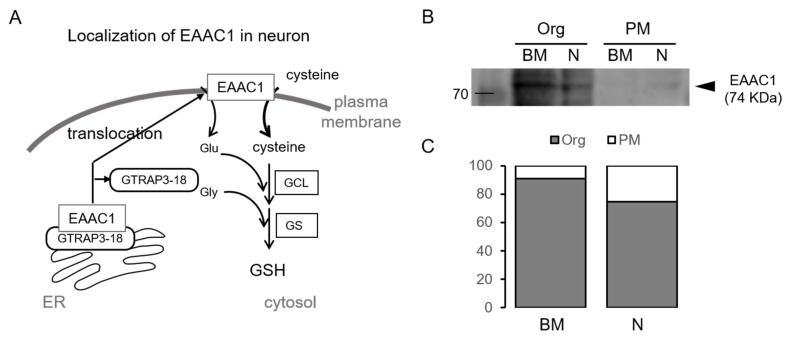
Subcellular localization of EAAC1 in BMSCs and NICD-3F BMSCs. (**A**) Schematic representation of EAAC1 trafficking in neuronal cells. Glutamate transporter-associated protein 3-18 (GTRAP3-18) retains EAAC1 in the endoplasmic reticulum (ER) and limits its plasma membrane trafficking. Translocation of EAAC1 to the plasma membrane promotes cysteine uptake, the rate-limiting step for GSH synthesis, catalyzed by glutamate-cysteine ligase (GCL) and glutathione synthetase (GS). (**B**) EAAC1 protein distribution in organelle-enriched membrane fraction (Org) and plasma membrane fraction (PM) fractions from BMSCs (BM) and NICD-3F BMSCs (N). Org and PM fractions obtained from equal amounts of starting material were subjected to Western blot analysis. (**C**) Quantification of EAAC1 subcellular localization. Approximately 9% of membrane-associated EAAC1 localized to the PM in BMSCs, whereas ~26% localized to the PM in NICD-3F BMSCs.

EAAC1 is known to be predominantly localized in an intracellular pool under basal conditions [16] (Figure 3A). Because EAAC1-dependent cysteine uptake differed between BMSCs and NICD-3F BMSCs (Figure 2), we examined whether differences in the subcellular localization of EAAC1 accounted for this observation. Equal amounts of starting material were fractionated into cytosolic, organelle-enriched membrane fraction (Org), and plasma membrane fraction (PM) (Figure S2A). Na^+^/K^+^-ATPase confirmed PM enrichment (Figure S2B), whereas GAPDH validated cytosolic enrichment and loading equivalence (Figure S2C). Western blot analysis revealed that most of the EAAC1 protein was localized in the Org fraction in both cell types (Figure 3B). However, quantification showed that only ~9% of total membrane-associated EAAC1 localized to the PM in BMSCs, while ~26% localized to the PM in NICD-3F BMSCs (Figure 3C).

These findings indicate that EAAC1 plasma membrane translocation is markedly limited in BMSCs compared with NICD-3F BMSCs, consistent with the reduced cysteine uptake observed in BMSCs.

### 2.5. GTRAP3-18 Protein Levels in BMSCs and NICD-3F BMSCs

EAAC1 trafficking is negatively regulated by the ER-associated protein GTRAP3-18, which binds EAAC1 and restricts its surface delivery (Figure 3A) [18,19,20]. Although GTRAP3-18 is abundantly expressed in neurons, its expression and function in BMSCs have not been well characterized.

**Figure 4:**
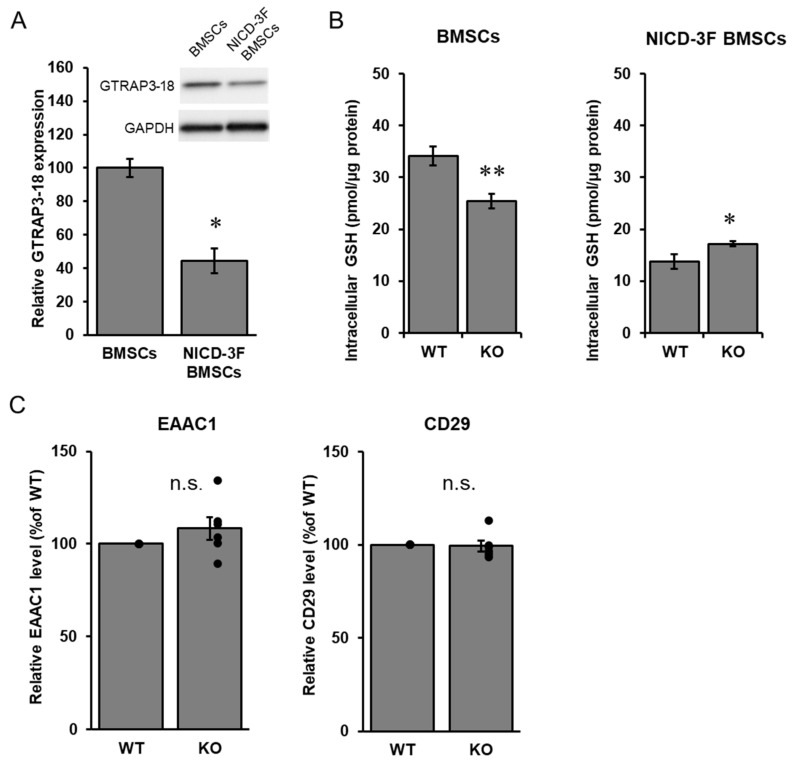
Effects of GTRAP3-18 on intracellular GSH levels and EAAC1 properties in BMSCs and NICD-3F BMSCs. (**A**) Western blot analysis of total GTRAP3-18 expression in BMSCs and NICD-3F BMSCs. Total GTRAP3-18 protein levels were significantly higher in BMSCs than in NICD-3F BMSCs (*n* = 4). (**B**) Intracellular GSH levels in BMSCs and NICD-3F BMSCs. Knockout (KO) of GTRAP3-18 reduced intracellular GSH levels in BMSCs but increased GSH levels in NICD-3F BMSCs compared with wild type (WT) control (*n* = 7–9). (**C**) Flow cytometric analysis of cell surface EAAC1 and the BMSC marker CD29 in WT and GTRAP3-18 KO BMSCs. The absence of GTRAP3-18 did not alter the surface abundance of either EAAC1 or CD29 (*n* = 6). For panels (**A**), *n* = 4 represents technical replicates derived from the same initial cell population. For panel (**B**), data were obtained from two independent mice, with 3–6 technical replicates per mouse (*n* = 7–9). For panel (**C**), data represent six independent biological replicates (*n* = 6). Data are presented as means ± s.e.m. Black circles represent individual data points. * *p* < 0.05; ** *p* < 0.01 compared with BMSCs or WT, as indicated. n.s., not significant.

Immunoblotting revealed that total GTRAP3-18 protein levels were substantially higher in BMSCs than in NICD-3F BMSCs (Figure 4A). The elevated GTRAP3-18 abundance in BMSCs could limit EAAC1 plasma membrane localization and thereby constrain cysteine uptake, whereas lower GTRAP3-18 levels in NICD-3F BMSCs would be consistent with enhanced EAAC1 surface availability and transport activity.

### 2.6. Effects of GTRAP3-18 Knockout on Intracellular GSH

To directly evaluate the functional contribution of GTRAP3-18, intracellular GSH levels were measured in BMSCs and NICD-3F BMSCs derived from GTRAP3-18 knockout (KO) mice. Unexpectedly, GSH levels were reduced by approximately 25.5% in GTRAP3-18 KO BMSCs, contrary to the prediction that loss of GTRAP3-18 would enhance EAAC1 trafficking and thereby increase GSH levels. In contrast, GSH levels were increased by approximately 25.5% in NICD-3F BMSCs lacking GTRAP3-18, consistent with enhanced EAAC1-dependent GSH synthesis in the neuron-like state (Figure 4B). Immunostaining showed that no apparent differences in neuronal marker expression were observed between WT and KO in NICD-3F BMSCs (Figure 1F and Figure S1).

Intracellular cysteine levels were not affected by deletion of GTRAP3-18 (Table S3). Likewise, flow cytometric analysis revealed no significant differences in surface expression of EAAC1 or CD29 between wild-type (WT) and KO BMSCs (Figure 4C). In addition, total EAAC1 protein levels remained unchanged between WT and KO cells in BMSCs, but were increased in NICD-3F BMSCs lacking GTRAP3-18 (Figure S3).

These findings suggest that GTRAP3-18 supports intracellular GSH maintenance in BMSCs through mechanisms independent of EAAC1-mediated cysteine uptake, while in NICD-3F BMSCs, GTRAP3-18 reduction facilitates EAAC1 trafficking and enhances GSH synthesis. In addition, the unchanged expression of the BMSC marker CD29 suggests that BMSC-like phenotypic characteristics may be preserved following the loss of GTRAP3-18.

## 3. Discussion

In this study, we sought to clarify the role of EAAC1 in regulating intracellular GSH levels in adult stem cells and in neuron-like cells regenerated from adult stem cells. Establishing a well-defined stem-to-neuron differentiation model was therefore essential for comparing EAAC1 function before and after neuronal lineage commitment. We first isolated BMSCs from mouse bone marrow and confirmed their identity based on the expected MSC phenotype—CD29^+^/Sca-1^+^/CD45^−^ (Figure 1A,B). A subset of cells with weaker or absent Sca-1 staining was also observed, indicating the presence of a heterogeneous cell population, which is a characteristic feature of MSCs [27]. In addition, spindle-shaped morphology and adipogenic differentiation potential further supported the MSC properties of the BMSC culture (Figure 1C,D). These results confirmed the successful preparation of a highly enriched and functional MSC population, providing a reliable starting point for downstream analyses.

Consistent with earlier reports, NICD drives BMSCs toward a neural stem/progenitor (NSC/NPC)-like phenotype in which multiple NSC/NPC-associated markers—such as GLAST, 3-PGDH, and nestin—are concomitantly induced [25]. Nestin, although originally identified as a neural stem cell marker, is also expressed in specialized subsets of mesenchymal stem/stromal cells, where it contributes to the maintenance of stemness properties and supports lineage plasticity [28]. In this study, we confirmed that our system reproduced the previously reported upregulation of nestin following NICD introduction, indicating that NICD-treated BMSCs acquire key features of a neural precursor-like state. Upon subsequent exposure to FSK, bFGF, and CNTF, these NICD-primed cells proceeded to differentiate into Tuj1^+^/NeuN^+^ neuron-like cells (NICD-3F BMSCs) (Figure 1F). However, a limitation of this study is that NICD-3F BMSCs do not exhibit characteristics of fully mature neurons. Although neuronal markers such as Tuj1 and NeuN were expressed, the weak expression of MAP2, together with the lack of functional neuronal validation, indicates that these cells remain in a partially differentiated, neuron-like state. Taken together, this differentiation system provides a tractable model for investigating how EAAC1 expression and function are altered during the transition from adult stem cells to neuron-like cells, with the important caveat that the cells represent an incompletely differentiated state rather than fully mature neurons.

We found that intracellular GSH levels were markedly higher in BMSCs than in NICD-3F BMSCs, whereas intracellular cysteine concentrations were similar (Figure 2A). This pattern resembles the well-established difference between glial cells and post-mitotic neurons, where glia consistently maintain substantially higher GSH levels than neurons [26,29], reflecting their greater antioxidant and detoxification capacity [30]. Although BMSCs exhibit a glia-like redox profile characterized by high intracellular GSH, the regulatory mechanisms that maintain GSH in undifferentiated BMSCs are still poorly understood. In particular, the identity of the cysteine uptake systems operating in BMSCs remains unresolved. Neurons depend almost exclusively on the high-affinity transporter EAAC1 for cysteine acquisition and GSH synthesis [11], whereas astrocytes primarily utilize the xCT (system Xc^−^) transporter [31]. Intriguingly, previous reports indicate that xCT inhibition alters redox homeostasis in MSCs [9] and xCT knockout reduces intracellular GSH level, resulting in the inhibition of osteogenesis in a MSC line [8], suggesting that cystine/cysteine handling contributes to their antioxidant capacity and osteogenesis; however, whether BMSCs additionally employ EAAC1—or whether EAAC1 becomes functional only after neural lineage commitment—has never been determined. In BMSCs, erastin treatment significantly reduced intracellular GSH levels, indicating that cystine transport pathways, including xCT, likely contribute to GSH homeostasis in these cells. In contrast, erastin also reduced intracellular GSH levels in NICD-3F BMSCs, with a more pronounced decrease observed in these cells. Given that NICD-3F BMSCs represent a partially differentiated, neuron-like state, this observation may reflect differences in cysteine transport mechanisms during neuronal differentiation. Alternatively, residual xCT activity or additional cystine transport pathways, as well as changes in GSH consumption, may contribute to the observed effects. The present study was designed to determine whether EAAC1 contributes to intracellular GSH regulation under the limited conditions of an in vitro cell culture model of neuronal differentiation-like conversion, rather than to establish the relative quantitative contributions of EAAC1 and xCT in vivo. In this context, our findings support the conclusion that EAAC1 participates in the regulation of intracellular GSH levels during neuronal differentiation-like conversion. However, we did not directly evaluate xCT expression or quantitatively compare EAAC1- and xCT-dependent transport activities. Therefore, the precise relative contributions of these transport systems to intracellular GSH regulation remain to be determined.

Next, we observed the same amount of total 74 kDa EAAC1, which is the mature form of the transporter protein [15,17], in BMSC and NICD-3F BMSCs. Despite similar total EAAC1 protein abundance, functional EAAC1-dependent cysteine uptake differed markedly between the two cell types. NICD-3F BMSCs consumed extracellular cysteine more efficiently than BMSCs, and EAAC1 inhibition with LAβHA reduced intracellular GSH only in NICD-3F BMSCs (Figure 2C,D). The absence of decreased intracellular cysteine following EAAC1 inhibition in NICD-3F BMSCs suggests that intracellular cysteine remains a small, rapidly utilized metabolic pool, tightly coupled to GSH synthesis. These results indicate that EAAC1 contributes to GSH synthesis after neuron-like differentiation, whereas BMSCs maintain high GSH levels through EAAC1-independent mechanisms.

To reconcile the discrepancy between comparable EAAC1 protein levels and differing cysteine uptake activities (Figure 2), we examined EAAC1 subcellular localization. As illustrated in Figure 3A, EAAC1 activity is regulated by trafficking, particularly through retention in the ER by GTRAP3-18 [18]. Our fractionation results showed that EAAC1 was predominantly localized in organelle-associated membranes in both BMSCs and NICD-3F BMSCs, consistent with previous reports that most EAAC1 resides in an intracellular pool under basal conditions [16]. Notably, NICD-3F BMSCs exhibited a higher fraction of EAAC1 at the plasma membrane (~26%) than BMSCs (~9%), suggesting increased membrane recruitment after neuron-like differentiation.

Although membrane translocation alone does not fully explain the magnitude of the difference in cysteine uptake activity, the increased plasma membrane localization in NICD-3F BMSCs likely contributes to their higher EAAC1-dependent cysteine transport. In neurons, protein kinase C activation doubles surface EAAC1 expression [16], indicating that EAAC1 can be rapidly mobilized from intracellular pools. Our findings suggest that BMSCs also maintain an intracellular reservoir of EAAC1 that may be mobilized under specific stimuli. Identifying the signaling pathways that regulate EAAC1 trafficking in BMSCs and during neuron-like differentiation will be important for understanding how cysteine uptake is controlled along the stem-to-neuron transition.

One of the major negative regulators of EAAC1 trafficking to the plasma membrane is the ER-associated protein GTRAP3-18, which binds EAAC1 and restricts its surface delivery (Figure 3A). In this study, GTRAP3-18 expression was markedly higher in BMSCs and substantially lower after neuron-like differentiation. This expression pattern corresponds well with the subcellular localization of EAAC1, in which EAAC1 surface translocation was limited in BMSCs and increased in NICD-3F BMSCs. Therefore, differential GTRAP3-18 expression likely contributes to reduced EAAC1 membrane localization in BMSCs and enhanced localization in the neuron-like state.

Loss of GTRAP3-18 would be expected to increase EAAC1 trafficking and elevate intracellular GSH levels (Figure 4A). However, in BMSCs, GTRAP3-18 deficiency paradoxically decreased intracellular GSH. In contrast, GTRAP3-18 knockout increased GSH in NICD-3F BMSCs, consistent with the canonical neuronal mechanism in which reduced GTRAP3-18 enhances EAAC1-dependent cysteine uptake and GSH synthesis.

These opposing effects suggest that GTRAP3-18 plays fundamentally different roles in stem cells and neuron-like cells.

In NICD-3F BMSCs, GTRAP3-18 likely functions through its established mechanism—retaining EAAC1 in the ER—to regulate intracellular GSH levels. In BMSCs, however, this mechanism does not appear to be dominant. Instead, GTRAP3-18 deficiency lowered intracellular GSH, indicating a noncanonical role for GTRAP3-18 in redox maintenance in stem cells. Although the detailed mechanism remains unclear, previous studies have shown that loss of ARL6IP5 (GTRAP3-18) disrupts ER Ca^2+^ homeostasis and induces ER stress-mediated apoptosis [32]. More recent work indicates that GTRAP3-18 contributes to ER structural integrity [33]. Thus, GTRAP3-18 deletion may impair ER quality control, reduce ER phagy, inhibit the removal of misfolded proteins, and increase ER stress, collectively driving a reduction in intracellular GSH in BMSCs.

Other factors may also contribute to the differential regulation of EAAC1 activity. GTRAP3-18 interacts with several trafficking regulators, including RTN2B, a reticulon family protein that facilitates EAAC1 export from the ER [17], and sorting receptor SorCS2, which sustain EAAT3/EAAC1 in Rab11^+^ recycling pool. [34]. Alterations in these pathways could further influence EAAC1 activity in a cell-type-dependent manner and remain to be investigated.

Finally, we cannot exclude the potential contributions of other cysteine/cystine transporters, such as xCT, which may participate in GSH regulation in BMSCs. Further investigation will be needed to fully elucidate how GTRAP3-18 coordinates EAAC1 trafficking, ER homeostasis, and redox balance across different stages of stem-to-neuron differentiation.

**Figure 5:**
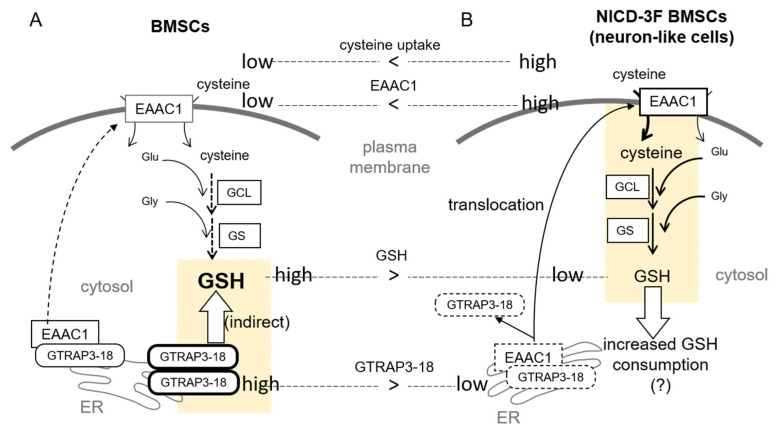
Proposed model of the roles of EAAC1 and GTRAP3-18 in regulating intracellular GSH levels in BMSCs and NICD-3F BMSCs. (**A**) In BMSCs, a large proportion of EAAC1 is retained within intracellular organelles, including the ER. Although GTRAP3-18 is highly expressed in BMSCs, the interaction between GTRAP3-18 and EAAC1 does not appear to play a major role in regulating intracellular GSH levels. Instead, GTRAP3-18 may contribute positively to GSH maintenance through mechanisms that are independent of EAAC1-mediated cysteine uptake. (**B**) In NICD-3F BMSCs, a greater proportion of EAAC1 localizes to the plasma membrane, resulting in enhanced cysteine uptake. Lower expression of GTRAP3-18 may facilitate EAAC1 trafficking to the plasma membrane and thereby may support intracellular GSH synthesis in the neuron-like state.

As proposed in Figure 5, GTRAP3-18 expression was high in BMSCs and markedly decreased following neuron-like cell differentiation, a pattern consistent with reduced EAAC1 plasma membrane localization in BMSCs and its enhancement in NICD-3F BMSCs. Although GTRAP3-18 is known to inhibit EAAC1 trafficking to the plasma membrane, the functional relationship between GTRAP3-18 and EAAC1 alone does not fully account for intracellular GSH levels in BMSCs. Loss of GTRAP3-18 did not promote EAAC1 membrane translocation in BMSCs and instead resulted in an unexpected reduction in intracellular GSH. In contrast, GTRAP3-18 deficiency in NICD-3F BMSCs was associated with increased GSH levels, consistent with the canonical neuronal mechanism in which reduced GTRAP3-18 enhances EAAC1-dependent cysteine uptake and subsequent GSH synthesis.

These findings indicate that the GTRAP3-18–EAAC1 interaction does not serve as a major regulatory axis for maintaining GSH homeostasis in BMSCs. Rather, GTRAP3-18 appears to contribute to intracellular GSH levels through EAAC1-independent mechanisms. Although the precise pathways remain to be elucidated, GTRAP3-18 has been implicated in maintaining ER structure, and its loss may disrupt ER homeostasis, leading to increased oxidative stress and decreased GSH levels in BMSCs.

Our results further suggest that GTRAP3-18 exerts broader biological functions beyond the retention of EAAC1 in the ER. In addition to modulating EAAC1-dependent cysteine uptake in neuron-like cells, GTRAP3-18 appears to play a critical role in sustaining redox balance in adult stem cells.

This study highlights a critical knowledge gap in the regulation of GSH metabolism. Because BMSCs possess the capacity to differentiate into neuron-like cells, elucidating how EAAC1 and GTRAP3-18 contribute to GSH regulation in BMSCs provides an opportunity to uncover previously unrecognized aspects of redox control during stem to neuron differentiation. Moreover, understanding the mechanisms governing GSH homeostasis in controlled neuronal differentiation systems derived from adult stem cells, including BMSCs, is expected to offer important insights that may inform the development of regenerative therapies for neuronal tissue repair based on adult stem cell transplantation.

## 4. Materials and Methods

### 4.1. Animals

C57BL/6J, GTRAP3-18 KO, and WT male or female mice aged 8 weeks were used for the isolation of BMSCs. C57BL/6J mice were purchased from Sankyo Labo Service Corporation (Tokyo, Japan). GTRAP3-18 KO and WT mice were described previously [23]. Mice were housed in a temperature-controlled environment (23 °C) under a 12 h light/dark cycle with food and water provided ad libitum. GTRAP3-18 KO mice were maintained on a C57BL/6J background. Wild-type littermates or age-matched C57BL/6J mice were used as controls. Male and female mice were not analyzed separately; however, experiments were performed using both sexes, with most experiments conducted using female mice. Neuron-like differentiation was confirmed in both sexes, and no obvious sex-dependent differences were observed. All procedures were approved by the Animal Ethics Committee of Teikyo University School of Medicine (Approval No. 16-005 and No. 23-009) and conducted in accordance with institutional guidelines.

### 4.2. Pharmacological Agents and Chemicals

L-aspartic acid β-hydroxamate (LAβHA) and Dulbecco’s Alpha Modified Eagle’s Medium (α-MEM) were obtained from Sigma Aldrich (St. Louis, MO, USA). Erastin was from Cayman Chemical (Ann Arbor, MI, USA). MesenCult Expansion Kit. (Mouse) and MesenCult Adipogenic Differentiation Kit (Mouse) were obtained from STEMCELL Technologies (Vancouver, BC, Canada). Fetal bovine serum (FBS) and 10,000 U/mL penicillin-streptomycin were from Gibco (Grand Island, NY, USA). Murine Recombinant bFGF and rat CNTF were from PeproTech (Cranbury, NJ, USA). FSK was from FUJIFILM Wako Pure Chemical (Osaka, Japan). Accutase was from Nacalai Tesque, Inc. (Kyoto, Japan). 4-fluoro-7-sulfamoylbenzofurazan (ABD-F) was from Dojindo (Kumamoto, Japan).

ABD-F was dissolved in methanol and diluted in borate buffer (pH 8.0) to 0.5 mM. LAβHA was dissolved in phosphate-buffered saline (PBS) to 100 mM. Erastin was dissolved in dimethyl sulfoxide to 2mM. FSK was dissolved in ethanol to 10 mM. These stock solutions were stored at −25 °C. bFGF and CNTF were dissolved in 1% bovine serum albumin in PBS to 10 µg/mL and stored at −80 °C.

### 4.3. Isolation of BMSCs

Crude bone marrow cells containing BMSCs were collected from femurs and tibias of each mouse by flushing with α-MEM supplemented with 10% FBS and 50 U/mL penicillin-streptomycin. Cells were passed through a 70 µm nylon mesh, pelleted at 250× *g* for 10 min, and resuspended in the culture medium, which consisted of MesenCult basal medium supplemented with 100 mM glutamine, MesenSupply, and MesenPure (MesenCult Expansion Kit, Mouse, Catalog #05513). Cells were seeded at 3–5 × 10^4^ cells/0.13 mL/cm^2^ and maintained for 7 days at 37 °C under 5% CO_2_ and 5% O_2_ in a multi-gas incubator (MG71M; TITEC, Saitama, Japan) to promote BMSC colony formation. Colonies were harvested with Accutase, reseeded into vessels of the same size and expanded for 3 days. Cells were subcultured and used for experiments within 5 days. BMSC surface markers were analyzed by flow cytometry (see Section 4.8).

### 4.4. Adipogenic and Neurogenic Differentiation

After 7 days of culture, adipogenic differentiation of BMSCs was induced using a standard kit (MesenCult Adipogenic Differentiation Kit, Mouse, Catalog #05507), and adipocytes were visualized by Oil Red O staining. Images were acquired using a Cell Culture Microscope (CKX53; Evident Corporation, Tokyo, Japan).

Neuron-like differentiation was initiated by introducing the NICD to induce neural stem cell-like properties. The NICD expression plasmid (see Section 4.5) was electroporated into BMSCs using a NEPA21 Type II electroporator (NEPA GENE Co., Ltd., Chiba, Japan). A total of 1 × 10^5^ BMSCs and 10 µg plasmid DNA in FBS-free α-MEM were placed in electroporation cuvettes (EC 002S). Electroporation parameters were: poring pulse, 150 V (duration 5 ms; interval 50 ms; 2 pulses; decay 10%; polarity +); transfer pulse, 20 V (duration 50 ms; interval 50 ms; ±5 pulses; decay 40%; polarity ±). The efficiency of NICD electroporation was initially estimated to be approximately 30% in preliminary experiments. This condition was selected based on an optimal balance between transfection efficiency and cell viability, which was sufficient to reproducibly induce neuron-like differentiation under the experimental conditions. Following electroporation, cells were cultured in α-MEM with 10% FBS without antibiotics for 2 days at 37 °C under 5% CO_2_ in a CO_2_ incubator (MCO-19AIC; PHC Corporation, Tokyo, Japan). To generate neuron-like cells, NICD-BMSCs were then treated with three neurotrophic factors (3F) such as 5 µM FSK (cAMP activator), 10 ng/mL bFGF, and 10 ng/mL CNTF for 3–5 days (NICD-3F BMSCs) [25]. Neuron-like differentiation was assessed by immunocytochemistry (see Section 4.9).

### 4.5. Plasmids

The NICD fragment (nucleotides 5186–7671 of mouse Notch 1) bearing a C-terminal 3 × FLAG tag was PCR amplified and subcloned into Gateway pcDNA DEST47 (Thermo Fisher Scientific, MA, USA; #12281010) using pCMV NICD 3 × FLAG (RDB_14500) as template. The template plasmid was obtained from the RIKEN BioResource Center (BRC), which participates in the National Bio Resource Project of MEXT, Japan.

### 4.6. Cell Culture Experiments

BMSCs and NICD-3F BMSCs were first cultured for 3 days under their respective maintenance conditions. BMSCs were maintained in α-MEM supplemented with 10% FBS at 37 °C in an atmosphere of 5% CO_2_ and 5% O_2_, whereas NICD-3F BMSCs were cultured in α-MEM supplemented with 10% FBS and three neurotrophic factors at 37 °C in 5% CO_2_. After this initial culture period, intracellular cysteine and GSH levels were assessed.

Cysteine uptake was then examined by incubating the cells for 90 min in fresh α-MEM, which inherently contained 200 µM cysteine and thus provided a newly replenished cysteine supply. In a separate series of experiments to determine whether EAAC1 and xCT contribute to maintaining intracellular GSH levels, the medium was replaced with fresh α-MEM with or without 1.2 mM LaβHA, an EAAC1 inhibitor, or 10 µM erastin, an inhibitor of the cystine/glutamate antiporter xCT, and the cells were incubated for 90 min. All incubations were performed under the same atmospheric conditions used for each cell type during the initial culture (i.e., 5% CO_2_ and 5% O_2_ for BMSCs; 5% CO_2_ for NICD-3F BMSCs). To assess potential cytotoxic effects of LaβHA and erastin treatments, total protein yield per well was evaluated as an indirect indicator of cell number and viability. Under the present experimental conditions (90 min treatment), total protein content per well showed no substantial difference between inhibitor-treated and vehicle control groups. These data suggest that treatment with either inhibitor did not significantly affect cell viability within the time frame of the experiments.

The intracellular cysteine and GSH levels were analyzed by HPLC; detailed procedures are described in Section 4.7.

### 4.7. HPLC Analysis of Cysteine and GSH

Cells were washed with cold PBS and homogenized in 200 µL 0.1 M perchloric acid containing 50 mM disodium EDTA (EDTA-2Na). Culture medium was mixed 1:1 with 0.2 M perchloric acid containing 100 mM EDTA-2Na. Homogenates and mixtures were centrifuged at 20,000× *g* for 15 min at 4 °C; deproteinized supernatants were used for ABD-F derivatization and HPLC.

Cysteine and GSH were derivatized with ABD-F (0.5 mM in borate buffer; 100 mM H_3_BO_3_, pH 8.0) at 50 °C for 5 min after pH adjustment to 5.0–5.6 with 0.1 M NaOH/0.1 M sodium acetate. Reactions were stopped on ice by adding HCl to a final concentration of 30 mM. Aliquots (50 µL) were injected onto a reverse-phase column.

Analyses were performed on a Nexera X2 UHPLC system (Shimadzu, Kyoto, Japan) with a CBM 20A, LC 30AD, SIL 30AC, RF 20Axs and CTO 20AC. An Inertsil ODS-2 column (150 mm × 4.6 mm; 5 µm) (GL Sciences, Tokyo, Japan) with a guard column (10 mm × 4.0 mm; 5 µm) was used. A linear/stepwise gradient elution was performed using solvent A (50 mM potassium biphthalate, pH 4.0) and solvent B (80% acetonitrile in H_2_O) as follows: 2% B at 0–2.0 min, increased to 5% B at 4.0 min, then to 10% B at 7.5 min and held at 10% B until 16.0 min. The proportion of B was immediately returned to 2% at 16.01 min and maintained at 2% until 21.0 min. Flow rate was 1.0 mL min^−1^; column temperature, 40 °C. Detection used excitation at 380 nm and emission at 510 nm. Data were processed with LabSolutions (Shimadzu, Kyoto, Japan). Analyte concentrations were calculated from peak areas against external standards. Retention times for ABD-F-derivatized cysteine and GSH were 7.0 min and 9.1 min, respectively.

### 4.8. Flow Cytometry

For flow cytometric analysis, Accutase-treated BMSCs were collected and resuspended in Fc block-containing stain buffer (0.5% bovine serum albumin in PBS), then incubated with fluorochrome-conjugated antibodies against CD29 (PE Cy7), Sca-1 (APC) and CD45 (PE) at 4 °C for 60 min. After washing, cells were analyzed on a flow cytometer (FACSCanto II; BD Biosciences, NJ, USA). Cell surface EAAC1 levels were analyzed by flow cytometry using primary antibodies directed against the extracellular domain of EAAC1 and the appropriate fluorophore-conjugated secondary antibodies. Full details of all antibodies used for flow cytometry are provided in Table S4.

### 4.9. Immunocytochemistry and Western Blot

For immunocytochemistry, BMSCs, NICD-BMSCs, and NICD-3F BMSCs were cultured on poly-D-lysine-coated and fibronectin-coated coverslips, respectively, and subsequently fixed with 4% paraformaldehyde at room temperature for 15 min. BMSCs were incubated overnight at 4 °C with pre-labeled primary antibodies against the CD29 antibody (1:500) and Sca-1 antibody (1:500), along with DAPI for nuclear staining. Negative controls were processed in parallel without primary antibodies. After washing, cells were counterstained with DAPI. NICD-BMSCs were incubated overnight at 4 °C with primary antibodies against nestin (1:200). After washing, cells were incubated with the appropriate fluorescence-conjugated secondary antibodies and counterstained with DAPI. These images were acquired using a fluorescence microscope (All-in-One Fluorescence Microscope BZ-X800; KEYENCE CORPORATION, Osaka, Japan). NICD-3F BMSCs were incubated overnight at 4 °C with primary antibodies against neuronal markers Tuj1 (1:67), NeuN (1:200), and MAP2 (1:100). After washing, cells were incubated with the appropriate fluorescence-conjugated secondary antibodies and counterstained with DAPI. Images were acquired using a confocal laser microscope (A1; NIKON INSTECH CO., LTD., Tokyo, Japan) and a confocal quantitative image cytometer (CQ1; Yokogawa, Tokyo, Japan).

For Western blotting, the cultured cells were washed with cold PBS and homogenized with RIPA buffer [50 mM Tris-HCl (pH 7.2)/150 mm NaCl/1% NP-40/0.25% sodium deoxycholate/1 mM EDTA/1 mM PMSF/1 mM NaF/1 mM Na_3_VO_4_/5 µg/mL of leupeptin, pepstatin and aprotinin]. Each homogenate was left on ice for 30 min and then centrifuged at 13,200× *g* for 5 min at 4 °C. The supernatant was mixed with a loading buffer [final concentration at 62.5 mM Tris-HCl (pH 6.8), 10% glycerol, 4% sodium dodecyl sulfate (SDS), 5% 2-mercaptoethanol, 1% DTT and 0.002% bromophenol blue] and was denatured at 98 °C for 3 min and separated by SDS-polyacrylamide gel electrophoresis (SDS-PAGE). For EAAC1 and Na^+^/K^+^-ATPase separation, a 7.5% polyacrylamide gel was used. For GTRAP3-18 separation, 12.5% polyacrylamide gel was used. Proteins were transferred to polyvinylidene fluoride membranes using a semi-dry transfer system (HorizeBLOT; ATTO, Tokyo, Japan). Membranes were blocked with 5% skim milk in Tris-buffered saline with 0.1% Tween-20 (TBST) at room temperature for 1 h and then incubated overnight at 4 °C with anti-EAAC1 (1:1000), anti-GTRAP3-18 (1:1000), and anti-Na^+^/K^+^-ATPase (1:2000) antibodies, which were diluted using Western BLoT Immuno Booster (Takara Bio Inc., Shiga, Japan). Anti-GAPDH antibody (1:5000) was diluted in TBST and incubated under the same conditions without Immuno Booster. After three washes with TBST, membranes were incubated with HRP-conjugated anti-rabbit or anti-mouse IgG antibody (see Table S3) and developed with ECL Prime (Cytiva, Marlborough, MA, USA). Immunoblotting data were collected using a Luminograph I (ATTO Corporation, Tokyo, Japan), measuring emitted photons by chemiluminescence. Protein expression was then evaluated using CS Analyzer 4 software (ver. 2.2.3; ATTO). GAPDH served as a loading control because its expression was unchanged between BMSCs and NICD-3F BMSCs under equal total protein loading.

The sources, catalog numbers, and host species of all primary and secondary antibodies used in this study are listed in Table S4.

### 4.10. Statistical Analysis and Replicates

Statistical analysis was performed using JMP Student Edition version 19 (SAS Institute Inc., Cary, NC, USA). Due to the sample sizes and to avoid assumptions of normal distribution, comparisons between two groups (e.g., BMSCs and NICD-3F BMSCs) were conducted using the Mann–Whitney U test. A *p* < 0.05 was considered statistically significant.

In this study, both biological and technical replicates were used depending on the experiment. In experiments involving multiple animals, cells were independently isolated from different mice (biological replicates) and analyzed with multiple technical replicates per biological sample. The number and type of replicates for each experiment are specified in the corresponding figure legends.

## Data Availability

The original contributions presented in this study are included in the article/Supplementary Material. Further inquiries can be directed to the corresponding author.

## Supplementary Materials

The following supporting information can be downloaded at: https://www.mdpi.com/article/10.3390/ijms27125323/s1.

## Abbreviations

The following abbreviations are used in this manuscript:

ABD-F	4-Fluoro-7-sulfamoylbenzofurazan
BMSC	Bone marrow-derived mesenchymal stem cell
bFGF	Basic fibroblast growth factor
CD29	Integrin β1
CNTF	Ciliary neurotrophic factor
EAAC1	Excitatory amino acid carrier 1
ER	Endoplasmic reticulum
FBS	Fetal bovine serum
FSK	Forskolin
GCL	Glutamate-cysteine ligase
GSH	Glutathione
GS	Glutathione synthetase
GTRAP3-18	Glutamate transporter-associated protein 3-18
HPLC	High-performance liquid chromatography
KO	Knockout
MAP2	microtubule-associated protein 2
MSC	Mesenchymal stem cell
NICD	Notch intracellular domain
NICD-3F BMSC	Neuron-like BMSC generated by NICD induction with three neurotrophic factors
NSC/NPC	neural stem/progenitor
Org	Organelle-enriched membrane fraction
PBS	Phosphate-buffered saline
PM	Plasma membrane fraction
Sca-1	Stem cell antigen-1
Tuj1	βIII-tubulin
WT	Wild type
xCT	Cystine/glutamate antiporter (SLC7A11)
