# Supplementary material for: Lineage-Dependent Regulation of Glutathione Homeostasis by EAAC1 and GTRAP3-18 During Differentiation of Mesenchymal Stem Cells into Neuron-like Cells
Source: Int J Mol Sci. 2026 Jun 12;27(12):5323. doi: 10.3390/ijms27125323 (PMC13300276; doi:10.3390/ijms27125323)
Supplement: Supplementary file 1 [file ijms-27-05323-s001.zip › ijms-4285678-supplementary.pdf]

Supplementary Materials

Lineage-dependent Regulation of Glutathione Homeostasis by EAAC1 and GTRAP3-18 during Differentiation of Mesenchymal Stem Cells into Neuron-like Cells

Nobuko Matsumura, Wattanaporn Bhadhprasit, and Koji Aoyama*
Department of Pharmacology, Teikyo University School of Medicine, 2-11-1 Kaga, Itabashi, Tokyo 173-8605, Japan;
*Corresponding author. E-mail address: aoyama.koji.ez@teikyo-u.ac.jp; Tel: +81-3-3964-3793; fax: +81-3-3964-0602.

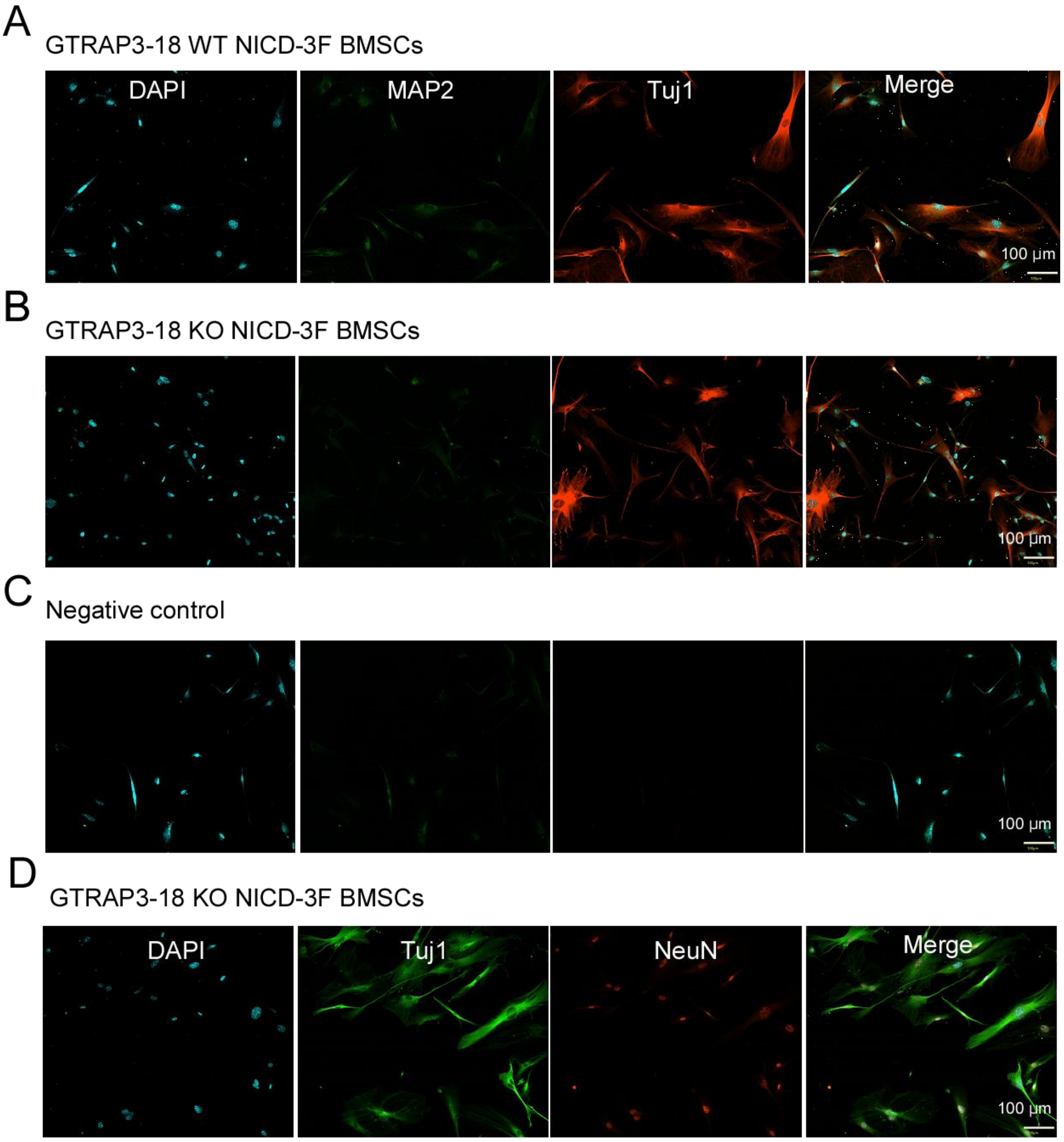

Figure S1. Immunofluorescence analysis of neuronal markers in WT and GTRAP3-18 KO NICD-3F BMSCs. Panels (**A**) and (**B**) show WT and GTRAP3-18 KO NICD-3F BMSCs stained for DAPI (blue), MAP2 (green), and Tuj1 (red). A negative control for panels (**A**) and (**B**) is shown in panel (**C**). MAP2 expression was weak in both WT and KO cells, consistent with an incompletely differentiated neuronal phenotype. Panel (**D**) shows GTRAP3-18 KO NICD-3F BMSCs stained for DAPI (blue), Tuj1 (green) and NeuN (red); images in panel (**D**) were acquired under lower excitation and exposure conditions compared with panel (**C**). Scale bars represent 100 μm.

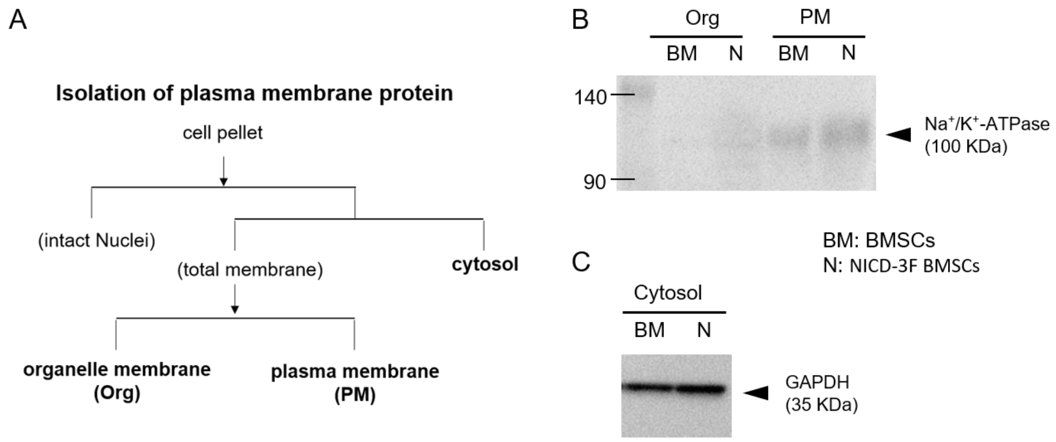

Figure S2. Subcellular fractionation of BMSCs and NICD-3F BMSCs. (**A**) Workflow for biochemical fractionation of cell lysates into cytosolic, organelle-enriched membrane (Org), and plasma membrane (PM) fractions. (**B**) Western blot showing Na^+/K^+-ATPase enrichment in PM fractions of both BMSCs (BM) and NICD-3F BMSCs (N). (**C**) GAPDH localization demonstrating cytosolic enrichment and verifying equal protein loading.

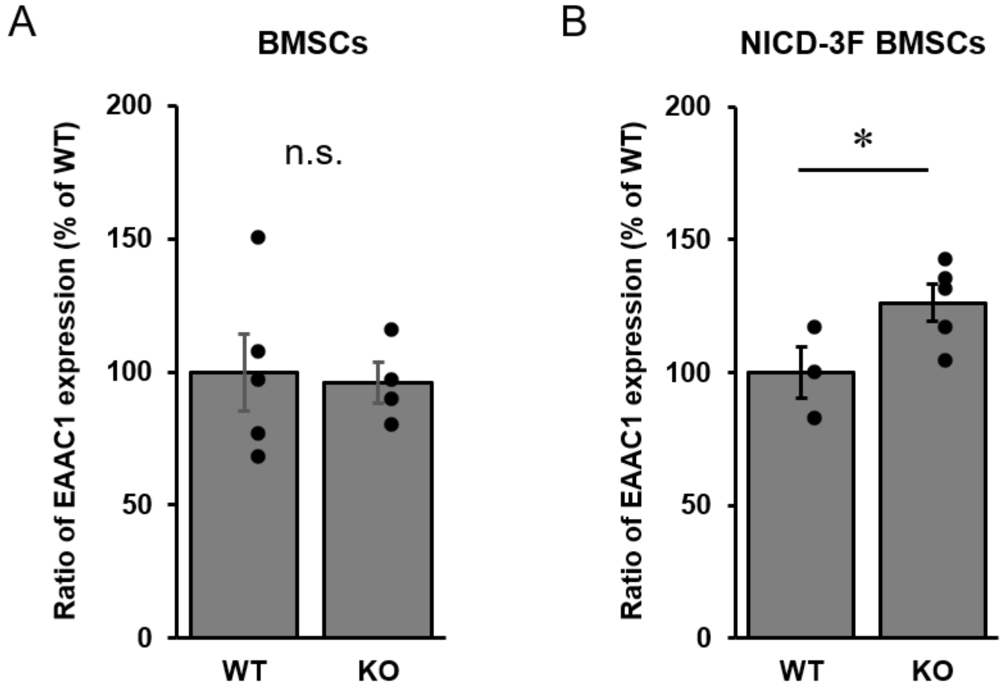

Figure S3. Total EAAC1 protein levels in WT and GTRAP3-18 KO cells. Total EAAC1 protein levels were unchanged in BMSCs but significantly increased in NICD-3F BMSCs lacking GTRAP3-18. (**A**) Total EAAC1 protein levels were not significantly altered between WT and KO BMSCs (n = 5 for WT and n = 4 for KO). (**B**) Total EAAC1 protein levels were significantly higher in KO than in WT NICD-3F BMSCs (n = 3 for WT and n = 5 for KO). For panel (**A**), data were obtained from two independent mice, with 1–4 technical replicates per mouse. For panel (**B**), data represent technical replicates derived from the same initial cell population. Data are presented as means ± s.e.m. Black circles represent individual data points. * $p < 0.05$ compared with BMSCs or WT, as indicated. n.s., not significant.

Table S1. Effects of an EAAC1 inhibitor on intracellular cysteine levels in BMSCs and NICD-3F BMSCs.

Intracellular cysteine levels were measured after treatment with vehicle (H_2O) or the EAAC1 inhibitor LAβHA (1.2 mM) under the same conditions as the GSH measurements in Figure 2D. No significant changes in cysteine levels were detected in either BMSCs or NICD-3F BMSCs (mean ± s.e.m.; n as indicated).

Cell type	Treatment	Cysteine (pmol / μg protein)	n	P value vs. vehicle (Mann–Whitney U test)
BMSCs	vehicle	0.43 ± 0.04	3	—
BMSCs	LAβHA	0.44 ± 0.06	3	0.50
NICD-3F BMSCs	vehicle	0.69 ± 0.04	6	—
NICD-3F BMSCs	LAβHA	0.74 ± 0.08	6	0.24

Table S2. Effects of an xCT inhibitor on intracellular cysteine levels in BMSCs and NICD-3F BMSCs.

Intracellular cysteine levels were measured after treatment with vehicle (dimethyl sulfoxide) or the xCT inhibitor erastin (10 μM) under the same conditions as the GSH measurements in Figure 2E. No significant changes in cysteine levels were detected in either BMSCs or NICD-3F BMSCs (mean ± s.e.m.; n as indicated).

Cell type	Treatment	Cysteine (pmol / μg protein)	n	P value vs. vehicle (Mann–Whitney U test)
BMSCs	vehicle	0.48 ± 0.01	7	—
BMSCs	Erastin	0.47 ± 0.03	7	0.50
NICD-3F BMSCs	vehicle	0.50 ± 0.03	3	—
NICD-3F BMSCs	Erastin	0.51 ± 0.02	3	0.33

Table S3. Effects of GTRAP3-18 knockout on intracellular cysteine levels in BMSCs and NICD 3F BMSCs.
Intracellular cysteine levels in WT and GTRAP3-18 KO cells are shown as mean ± s.e.m. No statistically significant differences in cysteine levels were observed (n.s.) in either BMSCs or NICD-3F BMSCs.

Cell type	GTRAP3-18 gene	Cysteine (pmol / µg protein)	n	P value vs. WT (Mann–Whitney U test)
BMSCs	WT	0.81 ± 0.08	9	—
BMSCs	KO	0.88 ± 0.10	8	0.33
NICD-3F BMSCs	WT	0.56 ± 0.11	7	—
NICD-3F BMSCs	KO	0.68 ± 0.05	8	0.22

Table S4. List of primary and secondary antibodies used for flow cytometry, immunocytochemistry, western blotting, and immunoprecipitation.
This table summarizes all antibodies employed in this study, including their dilution factors, suppliers, catalog numbers, and specific applications. For flow cytometry and immunocytochemistry, fluorophore-conjugated antibodies and filter settings are noted where applicable. For Western blotting, the corresponding secondary antibodies used for detection of each target protein are indicated. Abbreviations: HRP, horseradish peroxidase.

**Antibody name**	**Dilution**	**Company (Cat. no.)**	**Note**
Antibody for flow chemistry			
PE Rat Anti-Mouse CD45	1:23	BD Pharmingen (#553081)	—
CD29 (Integlin beta 1) Monoclonal Antibody PE-Cyanine7	1:50	ThermoFisher SCIENTIFIC (#25-0291-82)	—
Alexa Fluor® 647 Rat Anti-Mouse Ly-6A/E (Sca1)	1:23.	BD Pharmingen (#565355)	—

Mouse BD Fc Block	1:50	BD Biosciences (#553141)	—
Anti-SLC1A1 mouse monoclonal antibody (35-A9) (cell surface EAAC1)	1:50	Creative Biolabs (CBMAB-1109-YC) or Invitrogen (#32-1000)	—
Goat Anti-Mouse IgG H&L (Alexa Fluor® 647) preadsorbed	1:1000	abcam (ab150119)	For EAAC1 detection
Antibody for immunocyto chemistry			
CD29 (Integlin beta 1) Monoclonal Antibody PE-Cyanine7	1:50	ThermoFisher SCIENTIFIC (#25-0291-82)	Imaged under FITC filter settings
Alexa Fluor 647 Rat Anti-Mouse Ly-6A/E (Sca1)	1:50	BD Pharmingen (#565355)	Imaged under Cy5 filter settings
Anti-β-Tubulin III antibody produced in rabbit (Tuj1)	1:67	Sigma-Aldrich (T2200)	Detected with Alexa Fluor 488–conjugated secondary antibody (A-11008) for co-staining with NeuN. Detected with Alexa Fluor 594–conjugated secondary antibody (A11002) for co-staining with MAP2
Anti-RBFOX3/NeuN Mouse-Mono (1B7)	1:200	Novus Biologicals (NBP1-92693)	Detected with Alexa Fluor 594–conjugated secondary antibody (A11005).
Anti-Nestin antibody produced in rabbit	1:200	Sigma-Aldrich (N5413)	Detected with DyLight 488–conjugated secondary antibody (96899).

Anti-MAP2 antibody [EPR19691] Neuronal Marker Mouse IgG2a (Chimeric)	1:100	abcam (ab300645)	Detected with DyLight 488–conjugated secondary antibody (35503)
Goat anti-Rabbit IgG (H+L) Cross-Adsorbed Secondary Antibody, Alexa Fluor 488	1:1000	ThermoFisher SCIENTIFIC (A-11008)	For Tuj1 detection.
Goat anti-Mouse IgG (H+L) Cross-Adsorbed Secondary Antibody, Alexa Fluor 594	1:1000	ThermoFisher SCIENTIFIC (A11005)	For NeuN detection.
Goat Anti-Rabbit IgG H&L (DyLight 488) preadsorbed	1:1000	abcam (ab96899)	For Nestin detection.
Goat anti-Mouse IgG (H+L) Cross-Adsorbed Secondary Antibody, DyLight 488	1:1000	ThermoFisher SCIENTIFIC (35503)	For MAP2 detection.
Goat anti-Rabbit IgG (H+L) Cross-Adsorbed Secondary Antibody, Alexa Fluor 594	1:1000	ThermoFisher SCIENTIFIC (A11012)	For Tuj1 detection.
Antibody for Western blotting			
Anti-EAAT3 (EAAC1) antibody	1:1000	Alomone labs (AGC-023)	Detected with goat F(ab')$_2$ anti-rabbit IgG (HRP) (ab6112).
Anti-alpha 1 Sodium Potassium ATPase antibody	1:2000	abcam (ab7671)	Detected with anti-mouse IgG-HRP (AP181P).
Anti-Arl6ip5 polyclonal antibody (GTRAP3-18)	1:1000	Abnova (PAB8470)	Detected with goat anti-rabbit IgG (HRP) (AT55690).
Anti GAPDH mouse monoclonal antibody, (Clone 1E6D9)	1:5000	Proteintech (60004-1-Ig)	Detected with anti-mouse IgG-HRP (AP181P).

Peroxidase-Conjugated Goat Affinity Purified Antibody to Rabbit IgG Fc	1:2000	MP Biomedicals (AT55690)	Used for GTRAP3-18 detection.
Goat anti-mouse IgG antibody, HRP conjugate, species adsorbed	1:10000	Sigma-Aldrich (AP181P)	Used for GAPDH and Na^+/K^+-ATPase detection.
Goat $F(ab')_2$ Anti-Rabbit IgG F(ab')2 (HRP) preabsorbed	1:2000	abcam (ab6112)	Used for EAAC1 detection.
